# An LRR/Malectin Receptor-Like Kinase Mediates Resistance to Non-adapted and Adapted Powdery Mildew Fungi in Barley and Wheat

**DOI:** 10.3389/fpls.2016.01836

**Published:** 2016-12-15

**Authors:** Jeyaraman Rajaraman, Dimitar Douchkov, Götz Hensel, Francesca L. Stefanato, Anna Gordon, Nelzo Ereful, Octav F. Caldararu, Andrei-Jose Petrescu, Jochen Kumlehn, Lesley A. Boyd, Patrick Schweizer

**Affiliations:** ^1^Pathogen-Stress Genomics, Leibniz Institute of Plant Genetics and Crop Plant Research (IPK)Stadt Seeland, Germany; ^2^Plant Reproductive Biology, Leibniz Institute of Plant Genetics and Crop Plant Research (IPK)Stadt Seeland, Germany; ^3^National Institute of Agricultural BotanyCambridge, UK; ^4^Department of Bioinformatics and Structural Biochemistry, Institute of Biochemistry of the Romanian AcademyBucharest, Romania

**Keywords:** pathogen recognition receptor, PRR, 3D model, co-evolution, *Hordeum vulgare*, *Triticum aestivum*, *Blumeria graminis*

## Abstract

Pattern recognition receptors (PRRs) belonging to the multigene family of receptor-like kinases (RLKs) are the sensing devices of plants for microbe- or pathogen-associated molecular patterns released from microbial organisms. Here we describe *Rnr8* (for *Required for non-host resistance 8*) encoding HvLEMK1, a LRR-malectin domain-containing transmembrane RLK that mediates non-host resistance of barley to the non-adapted wheat powdery mildew fungus *Blumeria graminis* f.sp. *tritici*. Transgenic barley lines with silenced *HvLEMK1* allow entry and colony growth of the non-adapted pathogen, although sporulation was reduced and final colony size did not reach that of the adapted barley powdery mildew fungus *B. graminis* f.sp. *hordei*. Transient expression of the barley or wheat *LEMK1* genes enhanced resistance in wheat to the adapted wheat powdery mildew fungus while expression of the same genes did not protect barley from attack by the barley powdery mildew fungus. The results suggest that *HvLEMK1* is a factor mediating non-host resistance in barley and quantitative host resistance in wheat to the wheat powdery mildew fungus.

## Introduction

Plants recognize interacting beneficial or parasitic organisms via pathogen recognition receptors (PRRs) belonging to the highly complex and functionally diversified superfamily of receptor-like kinases (RLKs) that are also involved in plant development and abiotic-stress signaling (Macho and Zipfel, 2014). PRRs bind to microbe-associated molecular patterns, of with pathogen-associated molecular patterns (PAMPs) form a subgroup, and to damage-associated molecular patterns consisting of, for example, plant cell-wall degradation products. RLKs typically contain extracellular ligand-binding, transmembrane and cytoplasmic kinase domains. They are grouped into several sub-families depending on the presence of specific sequence motifs in their extracellular ligand binding domain, including leucine-rich repeats (LRRs), LysM, lectin-like, cysteine-rich or malectin domains, most of which bind to a corresponding class of ligands (Wu and Zhou, 2013). Upon ligand binding and activation, RLKs phosphorylate themselves or substrate proteins, which triggers signaling cascades such as the interacting mitogen-associated protein kinase kinase kinases cascade and leads to the execution of developmental or defense-related programs including the PAMP-triggered immunity (PTI) pathway (Jones and Dangl, 2006).

PAMP-triggered immunity is probably the underlying mechanism of a good number of cases of quantitative host resistance and of non-host resistance providing race-non-specific and durable disease resistance that ranges from partial to complete in the case of quantitative host resistance and non-host resistance, respectively (Kou and Wang, 2010; Fan and Doerner, 2012). Quantitative host resistance is usually less efficient than non-host resistance probably because pathogen-derived effector molecules (secreted proteins or non-proteinaceous small molecules) inhibit defense responses or stimulate pathogen accommodation by the host, which is referred to as effector-triggered susceptibility (ETS) (Deslandes and Rivas, 2012; Wawra et al., 2012; Giraldo and Valent, 2013). One of the preferred models for the efficiency and durability of non-host resistance states that, after host speciation, lack of co-evolution leads to gradual erosion of effector functionality in terms of their capacity to manipulate factors for defense or pathogen accommodation of previous host(s).

The genome of the barley powdery mildew fungus *Blumeria graminis* f.sp. *hordei* (*Bgh*) has been proposed to encode a set of over 500 candidate secreted effector proteins (CSEPs) with yet mostly unknown function (Pennington et al., 2016). A small proportion of these candidate effectors was recently tested functionally, which led to the discovery of a small family of RNAse-like proteins that appears to be important for initial host invasion (Pliego et al., 2013). The genome of the wheat powdery mildew fungus *B. graminis* f.sp. *tritici* (*Bgt*) encodes an even larger set of 602 CSEPs, which were defined by similar criteria as those from *Bgh* (Wicker et al., 2013). Most of the *Bgh* CSEPs share close homologs (orthologs) in *Bgt*, which suggests functional conservation of a large proportion of the host-invasion machinery by the two closely related pathogens. Until present only very few CSEPs have been described that interact with host-encoded proteins (Zhang et al., 2012; Schmidt et al., 2014). Therefore, a more or less comprehensive picture of the host-invasion mechanisms of powdery-mildew fungi is still missing.

Here we validate *Rnr8* (for *required for non-host resistance 8*) and present structural and functional data of the encoded protein HvLEMK1, an RLK of the LRR/malectin-domain sub-family. *HvLEMK1* was discovered in a transient-induced gene silencing (TIGS) screen for candidate genes of non-host resistance in barley to the non-adapted wheat powdery mildew fungus (Douchkov et al., 2014). The gene was silenced in transgenic barley plants and also transiently expressed in barley and wheat epidermal cells that were challenge-inoculated by adapted or non-adapted powdery mildew pathogens. The results suggest an important role for *LEMK1* in non-host resistance of barley and in quantitative host resistance of wheat to the wheat powdery mildew fungus.

## Materials and Methods

### Plant and Fungal Material

For the TIGS and transient over-expression experiments 7-day-old seedlings of the *Bgh*-susceptible spring barley cv. Maythorpe were used. Stable transgenic barley plants of cv. Golden Promise were generated as described (Hensel et al., 2008). Transient over-expression in wheat was done by using 7-day-old seedlings of the *Bgt*-susceptible cv. Kanzler. Bombarded leaf segments or transgenic plants were inoculated with Swiss *Bgt* field isolate FAL 92315, or Swiss *Bgh* field isolate CH4.8 throughout the study.

### cDNA Cloning of Wheat LEMK1 Orthologs

Initial searches of the NCBI^1^ and RIKEN EST^2^ databases, using *HvLEMK1* as the query sequence identified the best match to be the full-length EST TPLB0008D17, with a nucleotide similarity of 94%. Using this sequence, primers were designed using the software CLC DNA workbench, targeting a region upstream of the predicted start codon and downstream of the predicted stop codon: TaLEMK1_FL1_f1, TaLEMK1_FL1_r1, TaLEMK1_FL1_f2, TaLEMK1_FL1_r2 (Supplementary Table S1). The cDNA synthesis was performed using SuperScript III (Invitrogen) and the specific primer TaLEMK1_FL1_r2 with a mixture of RNAs from the wheat cultivar Renan. Renan was inoculated with the adapted (*Bgt*) and non-adapted (*Bgh*) pathogens of powdery mildew to ensure high levels of expression of TaLEMK1 transcripts. RNA was extracted 24 h after inoculation. Long range PCR was performed using the “Expand Long Template PCR system” from Roche Co. and following the manufacturer’s recommendation for cDNA templates between 0.5 and 9 kb. DNA fragments of the desired length were gel purified and cloned into pGEMT-easy. Colonies where tested for *TaLEMK1* inserts by colony PCR. Plasmids with expected insert size where verified by sequencing. For allele comparison between wheat genotypes, seedlings (14 days after sowing) of the winter wheat varieties Arran, Brock, Cadenza, Pastiche, Vault, and Zebedee were inoculated with *Bgh* (isolate CH4.8), and RNA was extracted 24 h after inoculation. The Illumina, RNA-seq pair-end reads from each of the six varieties were assembled into contigs using a compiled hexaploid wheat reference sequence (TGAC, Norwich, UK). Transcripts were assembled using this reference sequence. *TaLEMK1.1* (clone 7) and *TaLEMK1.2* (clone 12) were used as query sequences in BlastN searches against the assembled transcripts of the six winter wheat varieties.

### TIGS and Transient Over-Expression

Transient-induced gene silencing constructs were generated and transferred by particle bombardment into leaf epidermal cells of 7-day-old barley seedlings as described (Douchkov et al., 2005). Leaf segments were inoculated 3 days after the bombardment with *Bgh* at a density of 180–200 conidia mm^-2^. Transformed GUS-stained epidermal cells as well as haustoria-containing transformed (susceptible) cells were counted 48 h after inoculation, and TIGS effects on the susceptibility index (SI) were statistically analyzed as described in (Spies et al., 2012). For transient (over)expression, the *HvLEMK1*-containing BAC clone HvMRXALLhA0027N11 was bombarded into leaf segments of barley cv. Maythorpe or wheat cv. Kanzler, followed by challenge inoculation with the corresponding adapted pathogen *Bgh* or *Bgt* at a density of 180–200 conidia mm^-2^ 4 h after the bombardment and microscopic assessment of SI 48 h after inoculation. For the verification of *HvLEMK1* transgene effects, a 17.4 Kb *HvLEMK1*-containing BAC sub-clone (GenBank Acc. KR610392) was excised as StuI/SphI fragment and inserted into SmaI/SphI sites of transient expression vector pIPKTA09. In addition, barley and wheat cDNAs were excised and inserted as NotI fragment into to the multiple cloning site of pIPKTA09 (Zimmermann et al., 2006). The resulting sequence-verified constructs were bombarded into barley or wheat as described for BAC clones. Relative SI to empty BAC clone or to empty pIPKTA09 was calculated, log(2) transformed and tested for statistically significant deviation from the hypothetical control value “0” by a 1-sample *t*-test, 2-tailed.

### Inoculation and Evaluation of Transgenic Plants

Barley cv. Golden Promise was transformed with pIPKb009_HvLEMK1 (Himmelbach et al., 2007) as described (Hensel et al., 2008). Phenotypic evaluation of *Bgh* and *Bgt* interactions was done microscopically on second, detached leaves of 12–14 day-old T1 plants placed on phytoagar plates (23,2 cm × 23,2 cm) and inoculated at a spore density of 30–40 conidia mm^-2^. Leaf segments inoculated with either *Bgh* or *Bgt* were incubated strictly separated from each other in order to prevent cross-contamination. Golden Promise azygous T1 segregants served as internal negative controls. Inoculated leaf segments were incubated for 48 h (*Bgh*) or 7 days (*Bgt*) followed by staining with Coomassie brilliant blue R 250 (Schweizer et al., 1993). The number of growing *Bgt* colonies/leaf area was counted manually under a standard light microscope at 100× magnification. In case of *Bgh*, colonies were counted using the HyphArea software (Baum et al., 2011). Because of variability of residual *Bgt* susceptibility of the azygous control plants between the different inoculation experiments, we normalized the number of *Bgt* colonies to the average number of *Bgt* colonies on the azygous control in the corresponding experiment. Statistics: For *Bgt* susceptibility, non-normally distributed values from transgenic- or azygous control plants (a combined pool of null-allelic segregant plants from all T1 families) were subjected to Mann–Whitney test (2-tailed) for significant differences from the wildtype control. Data were obtained in three independent inoculation experiments. In the case of *Bgh* infection, data were normally distributed and tested by non-paired *t*-test against azygous control plants (2-tailed). Azygous control plants were used here as control because they were significantly more susceptible than wildtype plants, for unknown reasons. Data were obtained in two independent inoculation experiments.

To determine *HvLEMK1* transcript amounts in transgenic plants, total RNA was isolated from the 4th leaf using the RNeasy Plant Mini Kit with on-column DNase digestion (Qiagen, Hilden, Germany). Two micrograms of total RNA from 11 to 18 transgenic T1 progeny plants per primary transformant, or from a total of 32 azygous segregants, were reverse-transcribed using iScript^TM^ cDNA Synthesis Kit (Bio-Rad Laboratories, Inc.). Transcripts of *HvLEMK1* were quantified in triplicates by using TaqMan probes in a reaction volume of 10 μL (Maxima Probe qPCR Mastermix;Thermo Fisher Scientific, Waltham, MA, USA). Amplification and detection of fluorescent signal was performed in three technical replicates per cDNA sample on a 7900 HT Fast Real-Time PCR system (Life Technologies/Applied Biosystems, Darmstadt, Germany). To determine transcript amounts of proposed non-host-resistance marker genes, plants were grown in 22.5 cm × 18 cm pots in a plant incubator (Panasonic, Hamburg, Germany) at 20°C constant temperature, 60% relative humidity and 16 h illumination (intensity level 5) provided by daylight fluorescent tubes. Twelve-day-old plants were inoculated with *Bgt* spores (20–30 conidia/mm^2^), and second leaves were collected at respective time points for RNA isolation. Primary leaves were used for the hygromycin assay to identify azygous plants. RNA isolation, cDNA synthesis and qRT-PCR were done as mentioned earlier except a BRYT Green^®^ dye (Promega Corporation, USA) based system was used instead of TaqMan probes. Primer sequences for all PCR reactions are provided in Supplementary Table S1. Thermal cycling conditions consisted of initial denaturation at 95°C for 10 min followed by 30 cycles of (95°C/15 s, 55°C/40 s, 72°C/35 s). Ubiquitin conjugating enzyme 2 (*HvUBC*, Acc. AY220735) was used as internal normalization standard. To quantify the transcript levels in each sample, a standard curve for each gene with a serial dilution series was made from pooled RNA samples with three technical replicates each. Transcript quantities were determined using the SDS.2.4 software (Life Technologies GmbH, Darmstadt, Germany). For both *HvLEMK1*- and PTI-marker transcript quantification, two independent biological replicates were analyzed using plants grown on different dates.

### Subcellular Localization of Fluorescent Proteins

For subcellular localization, the full-length sequence of *HvLEMK1* was N- and C-terminally fused in-frame to *yellow fluorescent protein* (*YFP*) gene in pIPKTA48 and pIPKTA49 vectors, respectively, using Gateway^®^ cloning technology (Thermo Fischer Scientific, New York, NY, USA) (Supplementary Figure S1; Supplementary Table S1). Resulting YFP-fusion constructs were transiently expressed in 7-day-old leaf segments of barley cv. Golden Promise by particle bombardment and examined after 24 h of incubation without *B. graminis* inoculation using confocal laser scanning microscopy. The plasma-membrane marker *aquaporin* (*AtPIP2A*) of *Arabidopsis thaliana* (plasmid pm-rk CD3-1007), or the endoplasmic-reticulum (ER) marker *Wall-associated kinase 2* (*AtWAK2*; plasmid ER-rk CD3-959), both fused to mCherry as described in Nelson et al. (2007), were mixed in an equimolar concentration and co-bombarded for co-localization experiments. For plasmolysis of epidermal cells, leaf segments were floated on 15% glycerol for 5–10 min, immediately prior to microscopy.

### Transcript Regulation

Seven-day-old barley plants of cv. Vada or wheat plants of cv. Renan were inoculated with *Bgh* or *Bgt*, and the abaxial epidermis of inoculated primary leaves or from non-inoculated control leaves was peeled at 6–74 h after inoculation, as described (Zellerhoff et al., 2010; Spies et al., 2012). Total, quality-controlled RNA was hybridized to Agilent Gene Expression 44K microarrays (design ID: 020599 for barley and 022297 for wheat; Agilent) as described (Chen X. et al., 2011). Single-channel array processing was utilized followed by data normalization with default parameters, and significant transcript-regulation events were determined by using GeneSpring GX (v11.5.1) software (Agilent Technologies, Waldbronn, Germany). Transcripts were assumed to be significantly regulated if *p*-values corrected for false-positive rate (FDR, Benjamini-Hochberg method) were smaller than 0.05 and if regulation factors between inoculated and corresponding control samples harvested in parallel exceeded 2.0. All quantile-normalized signal intensities of the analyzed candidate genes are shown in Supplementary Table S1, and the raw data from the corresponding array slides were deposited at ArrayExpress [Accession Nr. E-MTAB-2916 for barley and E-MTAB-3803 for wheat]. Array procedures followed MIAME guidelines throughout (Brazma et al., 2001).

For the analysis of PAMP-induced *TaLEMK1* expression, wheat leaf segments were placed into 2 ml tubes submerged in H_2_O and pre-infiltrated by vacuum three times for 45 s. Leaf segments were left to recover in the growth cabinet for 16 h to avoid gene expression response to water infiltration. After this time, water was replaced by fresh water (control) or 1 mg/ml chitin (Yaizu Suisankagaku Industry Co., Ltd). Samples were drained, snap-frozen in liquid N_2_ at different time points and stored at -80°C. RNA was extracted using Qiagen RNAeasy Plant kit followed by removal of genomic DNA by DNase Turbo DNA-Free (Ambion). For quantitative real-time PCR (qPCR) analysis, first-strand cDNA was synthesized from 1 μg of total RNA using the SuperScript III First Strand Synthesis System. Quantitative RT-PCR (RT-qPCR) analysis of *TaLEMK1, TaCMPG1*-like and *TaPub23*-like was conducted as described in Tufan et al. (2009) using gene-specific primers (Supplementary Table S1).

### Modeling of HvLEMK1 Protein

Domain delineation, LRR repeat delineation, sequence analysis, and molecular modeling of the three domains of HvLEMK1 protein core were performed as described in Sela et al. (2012) and Slootweg et al. (2013). For model refinement an iterative procedure consisting in global simulated annealing with harmonic restraints on backbone atoms found in definite secondary structure states, followed by model quality assessment with MetaMQAP (Pawlowski et al., 2013). In order to optimize the HvLEMK1-LRR scaffold the Joint Fragment Remote Homology Modeling procedure (Sela et al., 2012; Slootweg et al., 2013) had to be used. The resulting 3D model was brought within 2.8 Å RMSD by repeated rounds of local remodeling from an optimal polypeptide path and a GDT_TS score of 60 according to MetaMQAP.

Sequence propensity analysis resulted in the following template selection for the three globular domains of (a) HvLEMK1-LRR: the LRR domain from FLS2 (PDB code: 4MN8) with sequence identity = 31%, confidence = 99; (b) HvLEMK1-malectin: the malectin from *Xenopus laevis* (PDB code: 2KR2) with sequence identity = 22%, confidence = 100; and (c) HvLEMK1-kinase: the kinase domains from BAK1 (PDB code: 3TL8) with sequence identity = 45%, confidence = 100 and from IRAK1 (PDB code: 2NRU) with sequence identity = 40%, confidence = 100. In addition the Joint Fragment Remote Homology Modeling procedure (Sela et al., 2012; Slootweg et al., 2013) was used to optimize the scaffold for Rnr8-LRR domain generation.

## Results

### Structure and Conservation of HvLEMK1

**FIGURE 1:**
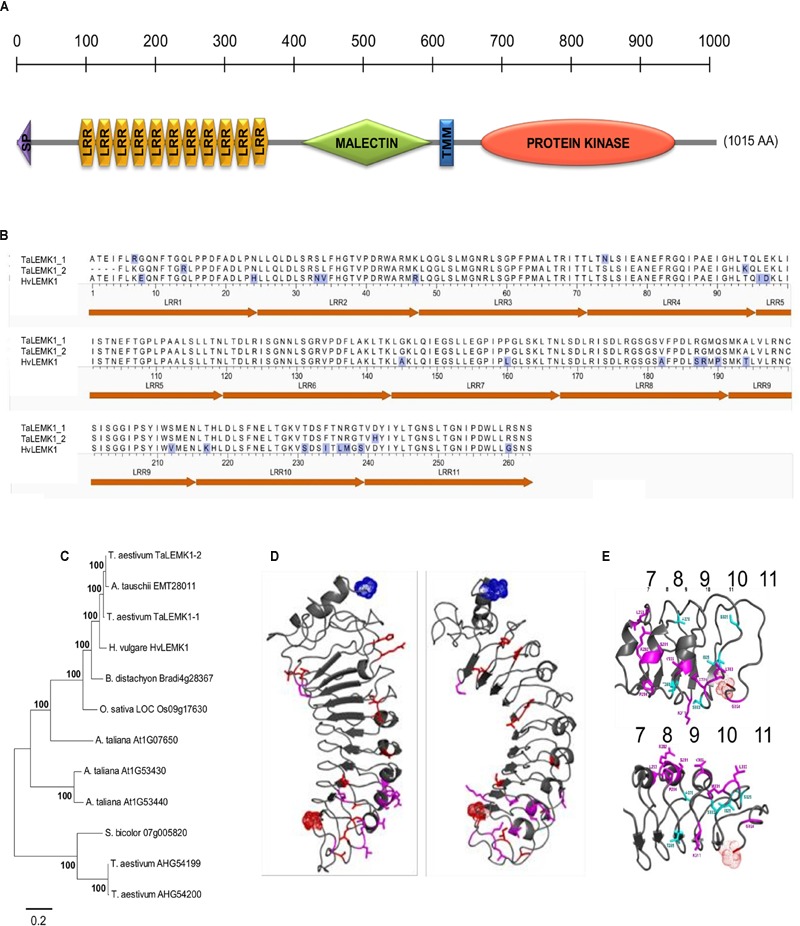
**Structure and conservation of *HvLEMK1*. (A)** Schematic representation of HvLEMK1 protein domains (drawn to scale). SP, signal peptide; TMM, transmembrane domain. **(B)** Amino-acid comparison of the LRR-repeat domain between barley and wheat LEMK1 proteins. Amino-acid exchanges are highlighted with blue shading. **(C)** Nearest-neighbor joining tree of full-length LEMK1-like protein sequences across monocot and dicot species. Most closely related homologs (putative orthologs) to HvLEMK1 from *Triticum aestivum* (bread wheat), *Aegilops tauschii* (wild wheat, donor of the D genome), *Brachypodium distachyon, Oryza sativa* (cultivated rice), *Sorghum bicolor*, and *Arabidopsis thaliana* were included. In addition, two more distantly related proteins from *T. aestivum* were included as outgroup. **(D)** Location of conservative and non-conservative amino-acid substitutions in red and magenta, respectively, among the 11 predicted LRRs. **(E)** Close-up of most variable repeats 7–11 between barley and wheat orthologs highlighting conservative and non-conservative amino-acid exchanges in light blue and magenta, respectively. The clustering of non-conservative site exchanges on the backside of the repeat structure is clearly visible in the lower part of the panel.

The *Rnr8* gene encodes for an LRR/malectin-domain RLK designated *Hv*LEMK1 that contains also a transmembrane and a C-terminal full-length protein-kinase domain (**Figure 1A**). Transient silencing of *HvLEMK1* was reported to weaken non-host resistance of barley to the wheat powdery mildew fungus *Bgt*, and therefore we searched for the presence of an orthologous protein in wheat, which is the natural host for *Bgt* (Douchkov et al., 2014). By PCR we identified two genes, *TaLEMK1_1* (Acc. KX529076) and *TaLEMK1_2* (Acc. KX529077), differing by five predicted amino acids in the LRR domain (**Figure 1B**; Supplementary Figure S2). Comparison of the full length sequences of *TaLEMK1* to the wheat chromosomes on URGI database^3^ revealed that the gene is located on the group 5 chromosomes, in syntenic position to the barley orthologous gene (Douchkov et al., 2014). Although exhibiting 94% nucleotide identify to *HvLEMK1*, evolutionary separation of the genus *Hordeum* and *Triticum* approximately 12 MY ago led to the accumulation of a total of 31 non-conservative amino-acid exchanges between the wheat and barley orthologs, which corresponds to approximately 3% of the protein (Supplementary Figure S2). Extending the search for close homologs of HvLEMK1 in more distantly related plants revealed the existence of HvLEMK1-like proteins in all analyzed species, and the conservation of the intron/exon structure suggested the existence of true orthologs (**Figure 1C**; Supplementary Figure S3). *TaLEMK1_1* and *TaLEMK1_2* were most closely related to genes on hexaploid wheat chromosome 5A and 5D, respectively, suggesting that they represent homeologs from the A and the D genomes of wheat. Because some of the non-*Triticum* plant species are not infected by any known powdery mildew fungus, *HvLEMK1* might function in sensing stress-related endogenous signals or highly conserved PAMPs present in different pathogens, or act as co-receptor in RLK complexes.

The putative ligand-binding LRR domain of HvLEMK1 has a homogeneous repeat length of 24. In order to put differences between the orthologous barley and wheat proteins into a structural context, a three-dimensional (3D) model was generated starting from the LRR domain of FLS2, a RLK protein from *Arabidopsis*, which contains repeats of the same length as HvLEMK1 (**Figure 1D**). Variable residues between HvLEMK1-LRR and TaLEMK1_2-LRR were mapped onto the 3D structure. The overall set of variable amino acids is shown here in red and magenta. Of these only those shown in magenta are mutations that might alter locally the structure and/or the interaction potential of the LRR domain. All these variable sites are clustered in the C-terminal region of the LRR domain pointing to a possible interaction surface with pathogen- or plant interaction partners. More specifically this cluster is located on the backside of repeats 8, 9, and 10 (**Figure 1E**). The malectin domain presents a conserved ordered beta core and a more unstructured N-terminal subdomain which hosts the lectinic site involved in glycan recognition of the malectin prototype protein in animals (Supplementary Figure S4A) (Chen Y. et al., 2011). Due to low sequence identity between HvLEMK1-malectin and the template protein, the 3D structure is still a rough model, with a current RMSD of 4 Å and a GDT_TS of ∼50. Finally, the HvLEMK1-kinase represented in Supplementary Figure S4B shows high sequence similarity with both plant RLK Bak1 and with human IRAK1, not only in the secondary structure patterns but also in the enzymatic active-site region. The model was brought within less than 3 Å RMSD from an optimal polypeptide path with an overall quality GDT_TS score of 58.

### Sub-Cellular Localization

**FIGURE 2:**
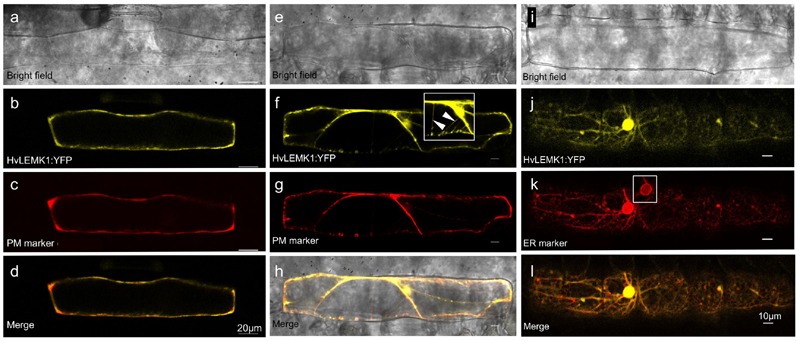
**Localization of C-terminal *Hv*LEMK1:YFP fusion protein in barley epidermal cells.** Barley leaf segments were co-bombarded with plasmids encoding HvLEMK1:YFP and the plasma-membrane localization marker pm-rk CD3-1007 (encoding aquaporin) or the endoplasmic reticulum-localization (ER) marker ER-rk CD3-959 (encoding *AtWAK2*) (Nelson et al., 2007). Confocal laser-scanning microscopy of non-inoculated cells was performed 24 h after the bombardment. **(a–d)** co-localization with the plasma-membrane marker. **(e–h)** plasma-membrane localization confirmed in plasmolysed cells. The inset in **(f)** corresponds to an over-exposed section of **(f)**, showing localization to Hechtian strands (arrowheads). **(i–l)** Co-localization with the ER marker. The inset in **(k)** corresponds to the cell nucleus imaged with reduced brightness in order to reveal the expected accumulation of the ER-marker at the nuclear border.

The encoded HvLEMK1 protein contains a transmembrane domain between malectin- and kinase domains, suggesting plasma-membrane localization, as has been shown for other RLKs. To verify this prediction, *HvLEMK1* cDNA was fused with its 3′-end to *YFP* and bombarded into barley epidermal cells. The resulting C-terminal fusion protein was tested for co-localization with an aquaporin:mCherry fusion protein that was used as plasma membrane marker, and with a AtWAK2:mCherry fusion protein used as marker for localization to the endoplasmic reticulum (ER) (**Figure 2**). This experiment revealed co-localization of HvLEMK1 with the plasma membrane marker in turgescent- and in plasmolyzed cells, and also in Hechtian strands (**Figures 2a–h**). In addition, we could co-localize a fraction of the protein with the ER marker (**Figures 2i–l**). It is known that plasma membrane-localized RLKs such as the malectin-domain protein Ferronia are retained in the ER, through which they are delivered to the cell periphery, if folding and quality-control chaperones in the ER are limiting or missing (Li C. et al., 2016b). Therefore ER-localization of part of the HvLEMK1:YFP protein pool may reflect overloading of the secretory system by transient over-expression under the control of the strong CaMV 35S promoter. However, because malectin is known to be ER-localized (Chen Y. et al., 2011) we cannot exclude a dual role of HvLEMK1 in both compartments. Finally, another fraction of HvLEMK1:YFP appeared to be localized to the nucleus, without a ring as seen with the mCherry ER marker (inset in **Figure 2k**) This unexpected result therefore awaits further functional examination.

### Regulation of LEMK1 Transcripts

**FIGURE 3:**
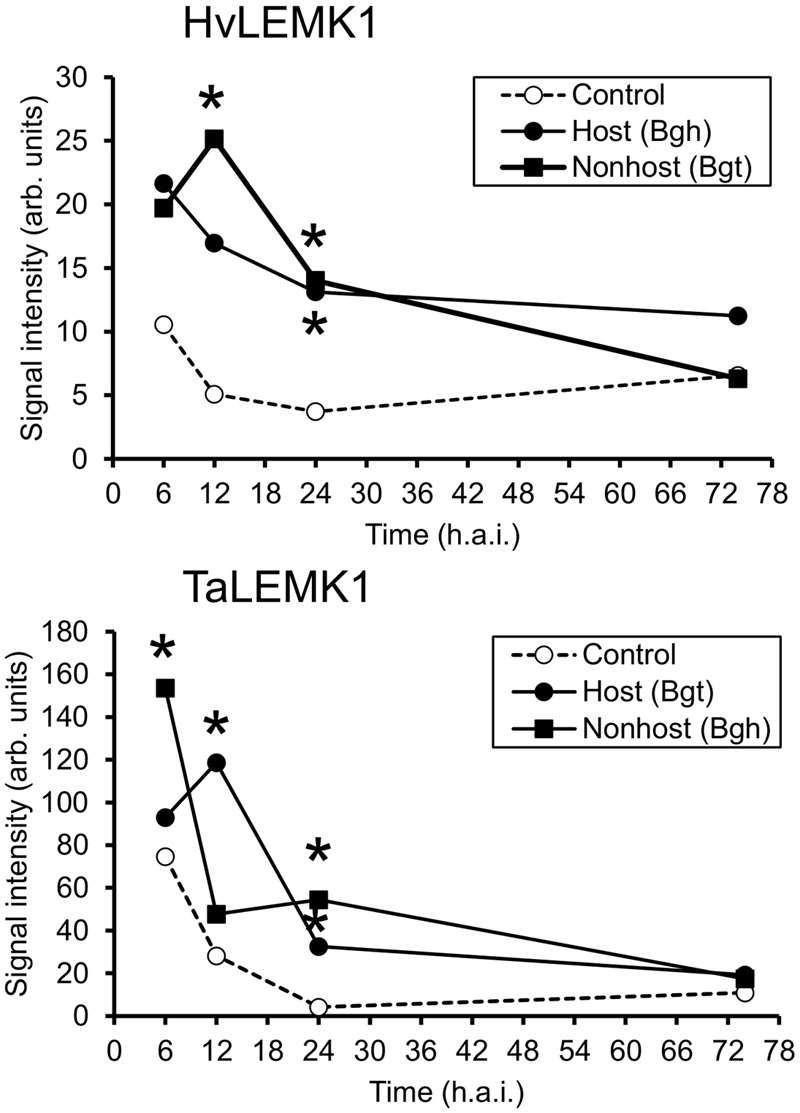
**Rapid transcriptional up-regulation of *LEMK1* in wheat and barley epidermis following inoculation with adapted or non-adapted powdery mildew fungi.** Data of three independent inoculation experiments were derived from Agilent 44K oligonucleotide microarrays, as described in section “Materials and Methods.” Asterisks indicate significant transcript regulation between inoculated and non-inoculated control plants (FDR-corrected *p*-value < 0.05). h.a.i., hours after inoculation.

Although RLKs are expected to exhibit a basal level of expression in order to fulfill their function in signal-perception, we often found their transcripts to be up-regulated upon pathogen attack of barley (Douchkov et al., 2014). We therefore interrogated two transcript-profiling datasets of leaf epidermal peels from barley and wheat challenged with *Bgh* and *Bgt*, which corresponds to a reciprocal set of host- and non-host interactions, by using the Agilent 44K Gene Expression Arrays of the two species. As shown in **Figure 3**, both *HvLEMK1* and *TaLEMK1* were up-regulated upon powdery-mildew attack. Interestingly, *Bgh* triggered maximum transcript levels at 6 h after inoculation in both plant species whereas the maximum response to *Bgt* attack was relatively delayed by 6 h. Therefore the different induction kinetics appears to be determined by the behavior of the respective pathogen (race) such as the kinetics of PAMP release, rather than by the host-vs. non-host status of barley or wheat.

### Transgenic Barley Silenced in *HvLEMK1*

**FIGURE 4:**
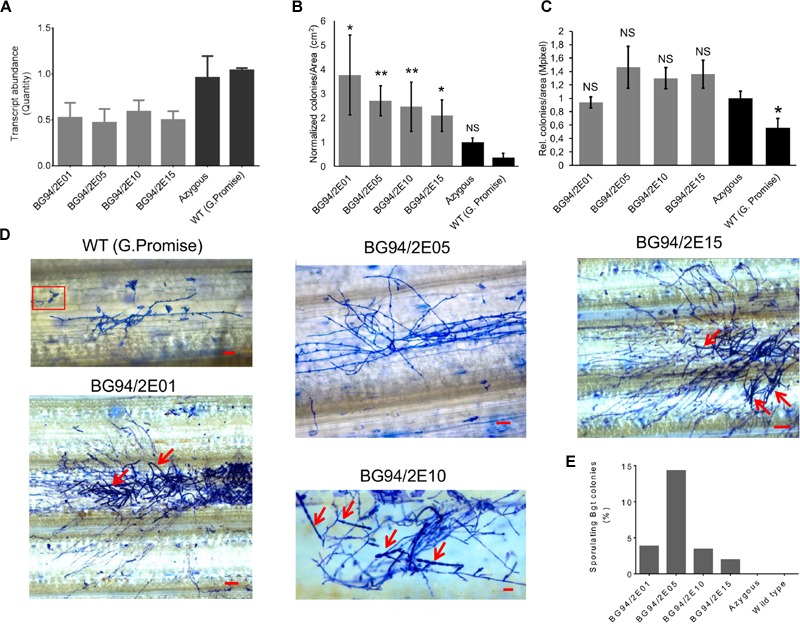
**Phenotype of RNAi transgenic T1 plants of barley with reduced *HvLEMK1* expression. (A)** RT-qPCR of *HvLEMK1* in selected T1 families. Mean values ± SE from two independent experiments (batches of plants sown on different dates, non-inoculated) are shown. **(B)** Reduced non-host resistance of transgenic events against *Bgt*. Mean values ± SE of manual microscopic colony counting 7 d.a.i. from three independent inoculation experiments, normalized to the mean value of the azygous control group, are shown. Statistical significance are indicated by asterisks. ^∗^*p* < 0.05, ^∗∗^*p* < 0.005. n, total number of analyzed plants per event. **(C)** No change in quantitative host resistance against *Bgh* of most of the T1 families shown in **(A)**. Mean values ± SE of automated microscopic colony counting by the HyphArea software at 48 h after inoculation (two independent inoculation experiments) are shown. Asterisk indicates statistical significance. ^∗^*p* < 0.05; n, total number of analyzed plants per T1 family; NS, not-significant. **(D)** Examples of enhanced colony growth including sporulation (red arrows) of *Bgt* on leaves of *HvLEMK1*-silenced T1 plants. Panel “WT (Golden Promise)” shows examples of frequently failed penetration (inside red frame) plus one of the rarely observed, non-sporulation *Bgt* micro-colonies. Scale bars = 50 μm. **(E)** Percentage of sporulating *Bgt* colonies (mean values from two independent experiments).

We stably transformed barley with an RNAi construct against *HvLEMK1* and identified 24 primary transgenic plants carrying intact versions of the silencing cassette. These were tested in the T1 generation for an altered interaction phenotype with *Bgt* (Supplementary Figure S5). T1 families with enhanced colony- or conidiospore formation of the non-adapted fungus in the first experiment were selected for subsequent tests in two more inoculation experiments, and for the quantification of target-transcript levels (**Figures 4A,B**). The susceptibility phenotype to *Bgt* could be confirmed in a statistically significant manner for four T1 families, and these also showed significant reduction of *HvLEMK1* transcript levels. On the other hand, we observed no difference of quantitative host resistance against *Bgh* in any of these four families (**Figure 4C**) suggesting no effect of *HvLEMK1* in quantitative host resistance. The microscopic inspection of *Bgt* development 7 days after inoculation (d.a.i.) of *HvLEMK1*-silenced leaves revealed the presence of colonies that, however, remained smaller than those of *Bgh* from parallel inoculations and were not visible to the naked eye (**Figure 4D**). Importantly, conidial chain formation of *Bgt* (highlighted by red arrows) was found in all four selected RNAi T1 families but never in the rare micro-colonies that were formed by *Bgt* on wildtype or azygous control plants (**Figures 4D,E**). We conclude that silencing of *HvLEMK1* resulted in a partial breakdown of non-host resistance to *Bgt*. Because the *HvLEMK1*-silenced plants showed no visible growth abnormalities or stress symptoms (Supplementary Figure S6) we assume that the *HvLEMK1* gene does not have essential developmental or housekeeping functions.

**FIGURE 5:**
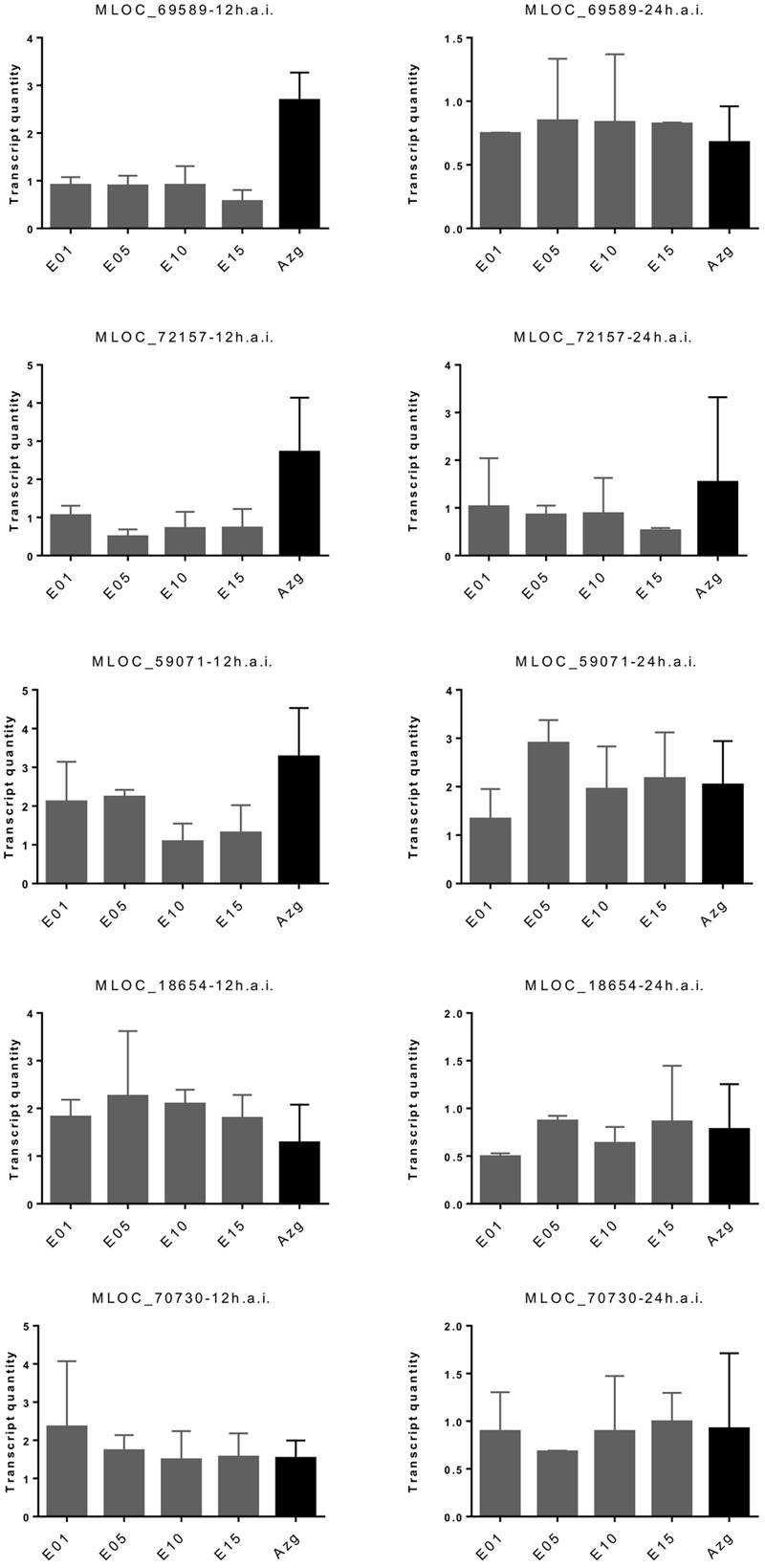
**Redued induction of proposed PTI marker genes in *HvLEMK1*-silenced barley plants.** Wildtype plants or selected transgenic T1 families were inoculated with *Bgt*, followed by RNA extraction 12 and 24 h after inoculation and quantification of transcript amounts by RT-qPCR. Mean values ± SE from two independent experiments (batches of plants sown on different dates) using a total of 10–14 transgenic plants are shown. Proposed protein functions: MLOC_69589, PR17-like secreted protein; MLOC_72157, SERK1-like RLK; MLOC_59071, putative leucoanthocyanidin dioxygenase; MLOC_18654, hypothetical protein; MLOC_70730, caffeoyl-CoA-*O*-methyltransferase.

We characterized transcriptional induction of five proposed marker genes of non-host resistance in the RNAi transgenic families. These genes were selected because their transcripts were more strongly induced at 6–12 h after inoculation in non-host resistant- compared to host susceptible interactions of barley and wheat with *B. graminis*. By contrast, they showed no differential induction between barley lines carrying the *Mla6* major R-gene or being mutated at the locus (*mla6*) (Supplementary Figure S9; Supplementary Table S2). As shown in **Figure 5**, three of the five candidate markers were less induced in *HvLEMK1*-silenced T1 populations at 12 h after *Bgt*-inoculation, compared to azygous segregant plants. At 24 h after inoculation the difference between azygous and transgenic plants had disappeared.

### Transient Expression of *LEMK1* Genes in Wheat and Barley

**FIGURE 6:**
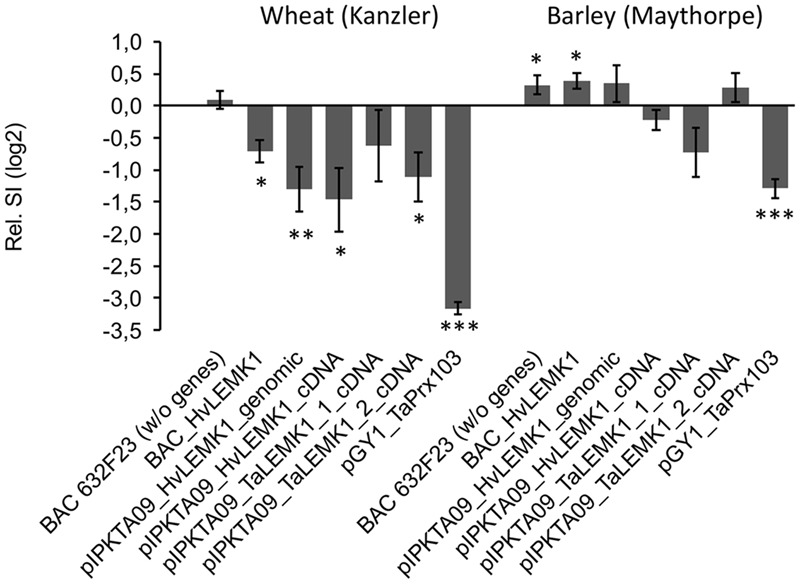
**Effect of transiently expressing *LEMK1* genes in barley and wheat on quantitative host resistance against adapted powdery mildew fungi.** Barley and wheat orthologs of *LEMK1* were transiently expressed in bombarded barley and wheat leaf segments, followed by inoculation with the adapted powdery mildew fungi *Bgh* and *Bgt*, respectively. In the case of *HvLEMK1*, the gene was expressed under the control of its own promoter by bombarding an *HvLEMK1*-containing BAC clone, and under the control of the CaMV 35S promoter as genomic BAC sub-clone (pIPKTA09_HvLEMK1_genomic) or full-length cDNA clone (pIPKTA09_HvLEMK1_cDNA). The corresponding wheat orthologs *TaLEMK1_1* and *TaLEMK1_2* were only expressed as full-length cDNA clones under the control of the CaMV 35S promoter (pIPKTA09_TaLEMK1_1_cDNA; pIPKTA09_TaLEMK1_2_cDNA). pGY1_TaPrx103 for over-expression of a pathogen-induced wheat class III peroxidase (Schweizer, 2008) and BAC632F23 containing no annotated genes served as positive and negative controls, respectively. SI values were normalized to the empty over-expression vector pIPKTA09 and log(2) transformed. Mean values ± SEM of 5–10 independent experiments (pGY1_TaPrx103 in barley, 21 experiments) are shown. ^∗^*p* < 0.05, ^∗∗^*p* < 0.01, ^∗∗∗^*p* < 0.0001 (1-sample *t*-test against the hypothetical control value “0,” 2-tailed).

We next tested the option that heterologous expression of the barley ortholog in wheat and *vice versa* might confer non-host-like resistance across species borders due to, for example, reduced functional suppression by one or several effectors from the corresponding non-adapted powdery mildew pathogen (Deslandes and Rivas, 2012; Feng et al., 2012; Mentlak et al., 2012). Bombardment of wheat-leaf segments with either a BAC clone carrying the *HvLEMK1* gene, the coding part of the gene under the control of the CaMV 35S promoter, or a full-coding sequence cDNA clone significantly enhanced resistance, as compared to a control BAC clone containing no annotated genes or to the empty over-expression vector pIPKTA9 (**Figure 6**). Transient over-expression of the two closely related *homoeo*-alleles in wheat revealed a protective effect of *TaLEMK1_2* comparable to the barley gene, whereas *TaLEMK1_1* showed no significant effect. One possible explanation to this observation is that the TaLEMK1_2 but not TaLEMK1_1 protein is free from functional suppression by a hypothetical *Bgt*-encoded effector. Attempts to transiently silence *TaLEMK1_1* and *TaLEMK1_2* for further clarifying their function in quantitative host resistance to *Bgt* and non-host resistance to *Bgh* failed because the bombarded wheat-leaf segments built up a strong resistance to *Bgt* within the minimum required incubation period of 2 days after bombardment that was required for effective TIGS. This bombardment-induced resistance, possibly due to mechanical stress, resulted in an average haustorial index of 0.006 from five independent experiments using the empty vector control. Stable transgenic RNAi lines of wheat would therefore be required to address the role of *TaLEMK1* genes in non-host resistance. In summary, at least the TaLEMK1_2 protein appears to quantitatively contribute to host resistance in wheat while its role in non-host resistance is currently unclear. In the light of the enhanced resistance caused by over-expression of endogenous *TaLEMK1_2* in wheat, the partial protection observed after transient expression of the heterologous *HvLEMK1* transgene might also be due to its over-expression rather than transgenomic functional complementation, even when bombarded as a BAC clone under the control of its own promoter.

## Discussion

Current models for non-host resistance suggest a range of defense-related proteins to be involved, in many cases belonging to the same families as those identified as major components of host PTI or ETI (Collins et al., 2003; Lipka et al., 2005; Stein et al., 2006; Sohn et al., 2012; Lee et al., 2014; Vega-Arreguin et al., 2014; Douchkov et al., 2016). It therefore appears plausible that non-host resistance in many instances is conferred by the robust execution of a host-defense program, which might be very diverse depending on the molecular, cellular, and physiological characteristics of a given non-host plant-pathogen interaction. A recent general model proposes that the recognition of non-adapted pathogens shortly after host speciation is mediated mostly by the robust combinations of NB-LRR-type effector recognition proteins triggering ETI whereas in more ancient non-host interactions, strong PTI responses are activated by PAMP-recognizing PRRs for the inhibition of which the non-adapted pathogen had lost functional effectors (Schulze-Lefert and Panstruga, 2011).

Here we propose LEMK1, an LRR/malectin-domain RLK, to mediate non-host resistance in barley and quantitative host resistance in wheat against the wheat powdery-mildew fungus *Bgt*. As suggested from the presence of an N-terminal transmembrane domain and from the localization of a *Hv*LEMK1:YFP fusion protein, HvLEMK1 appears to reside in the cell membrane probably transmitting an extracellular signal. It possesses 11 predicted LRRs, and this number of repeats corresponds, for example, to the R-protein encoded by the barley *Mla1* gene conferring resistance to *Bgh.* However, repeat number is lower than in many other reported R-proteins including wheat *Pm3b* for resistance to *Bgt* (Meyers et al., 2003; Yahiaoui et al., 2004). The malectin domain, which is related to the endoplasmic reticulum-localized, high mannose glycan-binding lectin malectin of animals (Galli et al., 2011), has been involved in RLK cleavage resulting in release from steric hindrance of heteroduplex formation and in fine tuning of RLK turnover (Lindner et al., 2012; Antolin-Llovera et al., 2014a,b). However, because the conserved GDPC motif required for cleavage of the malectin domain is missing from the predicted HvLEMK1 protein its function might be rather related to extracellular mannan-rich carbohydrate perception, as proposed for *Catharanthus roseus* RLK1-like kinases (CrRLK1Ls) including the fertilization and defense-related Ferronia protein (Lindner et al., 2012). It is interesting to note here that a highly active, secreted PAMP of *Bgt* was characterized as a glucomannan, with the mannan-residues being important for elicitor activity (Schweizer et al., 2000). Future biochemical work will have to address this possibility. Also, besides the possibility that HvLEMK1 recognizes an extracellular PAMP or a damage-associated molecular pattern, which would suggest it to act as PRR, it could function as co-receptor to PAMP receptors similar to the LRR/malectin RLK IOS1 recently characterized in *A. thaliana*, (Chinchilla et al., 2007; Niehl et al., 2016; Yeh et al., 2016). An analysis of allelic diversity of the LRR domain among different barley and wheat genotypes revealed a very high degree of intra-species conservation within each species (Supplementary Figure S7). Although this result might be strongly influenced by a breeders-driven selection for specific alleles, it supports the view that *LEMK1* is not engaged in a co-evolutionary arms race for pathogen-effector recognition but may mediate the recognition of more conserved (PAMP) signals or be a co-receptor to endogenous PRRs. This hypothesis is also in line with the observed inter-species variability in amino-acid sequences between barley and wheat, which might reflect the selection for optimal interaction with such conserved interaction partners in each species.

Further support for a role in PAMP-mediated signaling comes from transcript regulation data in wheat showing induction by chitin (Supplementary Figure S8), a well-established PAMP in many plant-pathogen interactions (Kaku et al., 2006). It is known that PRRs are transcriptionally up-regulated by PAMPs (New et al., 2015; Li B. et al., 2016a). The induction of *TaLEMK1* by chitin does not, however, qualify this LRK as chitin receptor because single PAMPs were found to induce more than one PRR (New et al., 2015). The observed, delayed accumulation of three of the five candidate markers in *HvLEMK1*-silenced T1 populations at 12 h after *Bgt*-inoculation, compared to azygous segregant plants, also suggest a role at the RLK in defense-regulated gene expression. At 24 h after inoculation the difference between azygous and transgenic plants had disappeared, which might indicate signal redundancy between different RLKs or the overriding of the RNAi effect by *HvLEMK1* transcript induction. The two non-affected transcripts from genes MLOC_18654 and MLOC_70730 might be regulated by an independent signaling pathway. In summary, the PAMP-mediated induction of *TaLEMK1* transcripts, and the *HvLEMK1*-dependent induction of non-host marker genes suggest that *LEMK1* is involved in two manifestations of PTI: quantitative host resistance and non-host-resistance (Fan and Doerner, 2012).

**FIGURE 7:**
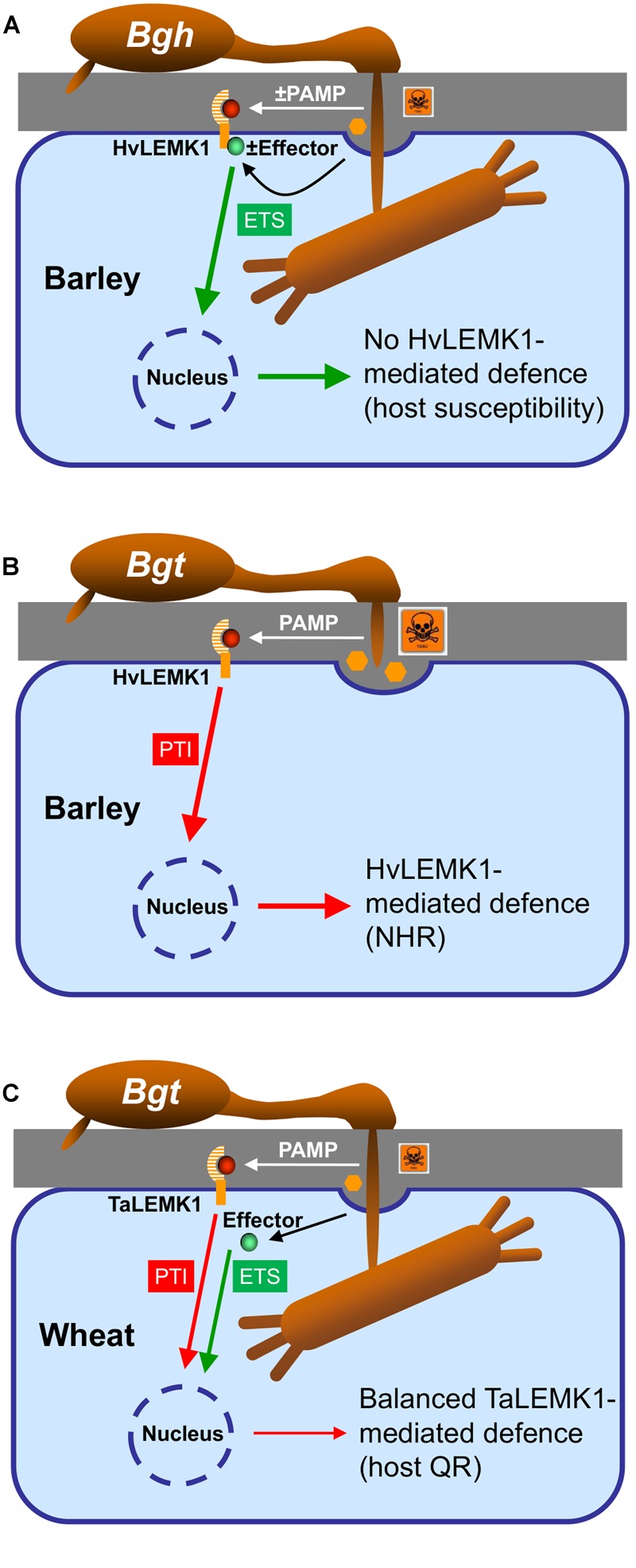
**Model of the role of LEMK1 in non-host resistance of barley and quantitative host resistance of wheat to *Bgt*. (A)** In the susceptible host interaction, *Bgh* either prevents or masks the release of a PAMP (highlighted by “±,” which otherwise would be recognized by HvLEMK1, or it releases an effector suppressing HvLEMK1-triggered PTI. **(B)** In the non-host-resistant interaction of barley with *Bgt*, HvLEMK1 recognizes the same/similar conserved PAMP without attenuation by an effector and triggers a strong PTI response. **(C)** During the interaction of wheat with *Bgt*, TaLEMK1 also recognizes the corresponding PAMP with high efficiency, but PTI is partially inhibited by other effector(s) from *Bgt*. **(A–C)** For simplicity the model only includes a proposed role of LEMK1 as PAMP receptor and ignores the possibility that it acts as co-receptor together with another RLK. In both scenarios, however, the outcome of the presence/absence of a PAMP or of a LEMK1-suppressing effector would be the same. NHR, non-host resistance; host QR, host quantitative resistance.

Transient over-expression of *TaLEMK1_2* as well as *HvLEMK1* enhanced resistance in wheat to *Bgt* but not in barley against *Bgh* suggesting that *Bgt* is more strongly affected by increased levels of the LEMK1 protein. Two possible explanations might apply to these different *LEMK1* effects in the two *B. graminis formae specialis*: *Bgh* but not *Bgt* might possess an effector controlling HvLEMK1 function, or the fungus might degrade or mask some PAMP molecule(s) as has been described before for other pathogens (van den Burg et al., 2004). Which of these possibilities that are summarized in the model shown in **Figure 7** are true remains to be further examined in the future.

*HvLEMK1* and *TaLEMK1_2* were discovered as factors mediating non-host resistance in barley to *Bgt* and quantitative host resistance in wheat to the same pathogen indicating that *Triticeae* species share components of quantitative host-and non-host resistance. This supports the idea that non-host resistance is an extremely strong manifestation of quantitative host resistance, which is difficult for a non-adapted pathogen to overcome because it would require incremental increases of pathogenicity or aggressiveness, which cannot be selected for in the absence of reproductive cycles on the non-host. Finally, the discovery and validation of non-host resistance components of barley may provide valuable leads for quantitative host resistance improvement by allelic optimization of orthologs in wheat, or by introgression across species borders by genetic engineering (Lacombe et al., 2010).

## Author Contributions

LB designed research and wrote the article. OC performed research (modeling). DD performed research (transgenic plant analysis). NE performed research (gene cloning and analysis). AG performed research (gene cloning and analysis). GH performed research (plant transformation). JK designed research. A-JP designed and performed research (modeling). JR performed research and wrote the article (gene cloning, transient expression, sub-cellular localization, transgenic plant analysis, transcript profiling). FS performed research (gene cloning and sequencing). PS designed research and wrote the article.

## Conflict of Interest Statement

The authors declare that the research was conducted in the absence of any commercial or financial relationships that could be construed as a potential conflict of interest.

## Footnotes

1 http://www.ncbi.nlm.nih.gov/

2 http://barleyflc.dna.affrc.go.jp/bexdb/

3 http://wheat-urgi.versailles.inra.fr/Tools/dbWFA
