# Supplementary material for: An LRR/Malectin Receptor-Like Kinase Mediates Resistance to Non-adapted and Adapted Powdery Mildew Fungi in Barley and Wheat
Source: Front Plant Sci. 2016 Dec 15;7:1836. doi: 10.3389/fpls.2016.01836 (PMC5156707; doi:10.3389/fpls.2016.01836)
Supplement: Supplementary file 3 [file Presentation_1.PDF]

frontiers

Supplementary Material

An LRR/malectin receptor-like kinase mediates resistance to non-adapted and adapted powdery mildew fungi in barley and wheat

Jeyaraman Rajaraman[1], Dimitar Douchkov[1], Götz Hensel[1], Francesca Stefanato[2], Anna Gordon[2], Nelzo Ereful[2], Octav F. Caldararu[3], Andrei-Jose Petrescu[3], Jochen Kumlehn[1], Lesley A. Boyd[2], and Patrick Schweizer*[1]

*** Correspondence:** Patrick Schweizer: schweiz@ipk-gatersleben.de

1 Supplementary Data

Supplementary Table S1: Sequences of PCR primers used in the study. Submitted as separate file (Excel).

Supplementary Table S2: Primary signal intensity values of *HvLEMK1* and *TaLEMK1* transcripts hybridized to Agilent Gene Expression 44K microarrays of barley and wheat. Submitted as separate file (Excel).

2 Supplementary Figures

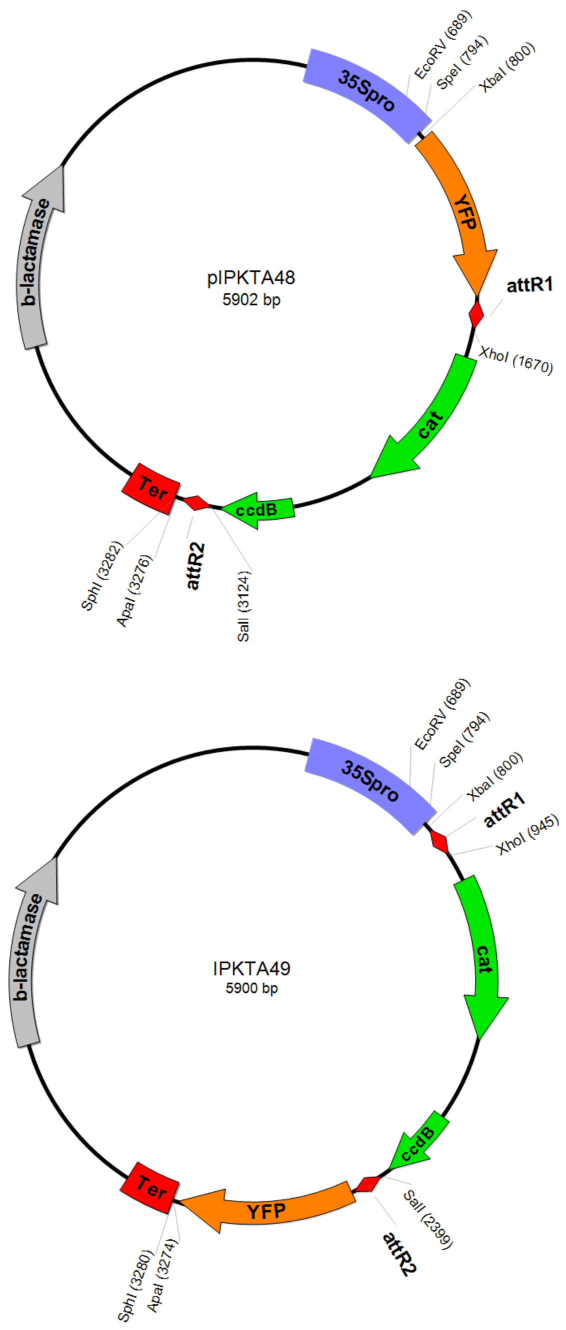

Supplementary Figure S1: Schematic diagrams of the vectors pIPKTA48 and pIPKTA49 for transient expression of YFP-tagged plant proteins. pIPKTA48 and pIPKTA49 result in N-terminal and C-terminal fusions, respectively.

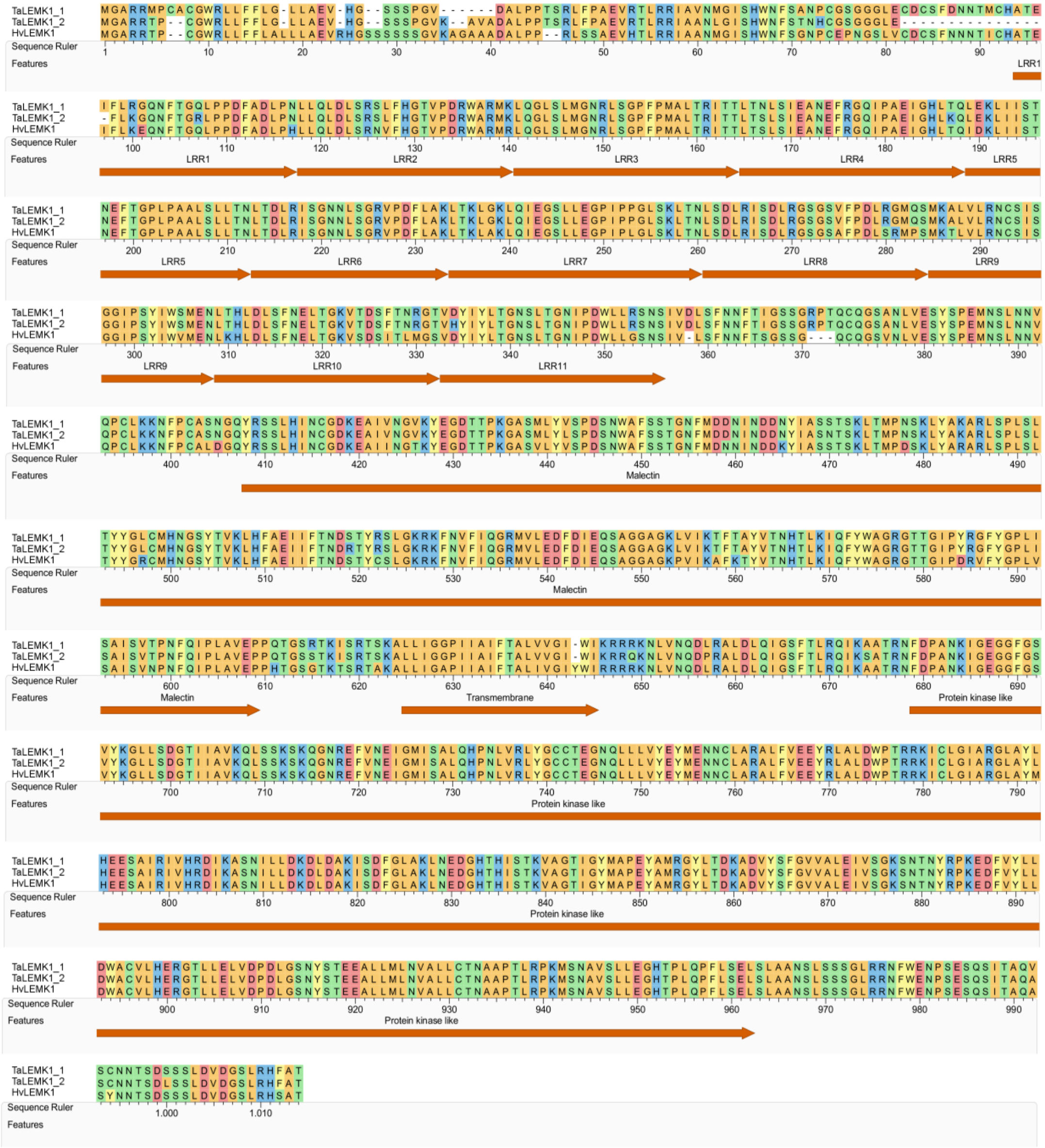

Supplementary Figure S2: Alignment of LEMK1 amino-acid sequences from cultivated barley (*H. vulgare* ssp. *vulgare*) and hexaploid wheat (*T. aestivum*).

Multiple alignment in Geneious software package with the following parameters: Geneious global alignment with free end gaps, cost matrix Blosum62, gap open penalty 12, gap extension penalty 3, two refinement iterations. Amino acid residues are coloured according to polarity. Annotated protein domains are highlighted below the alignment.

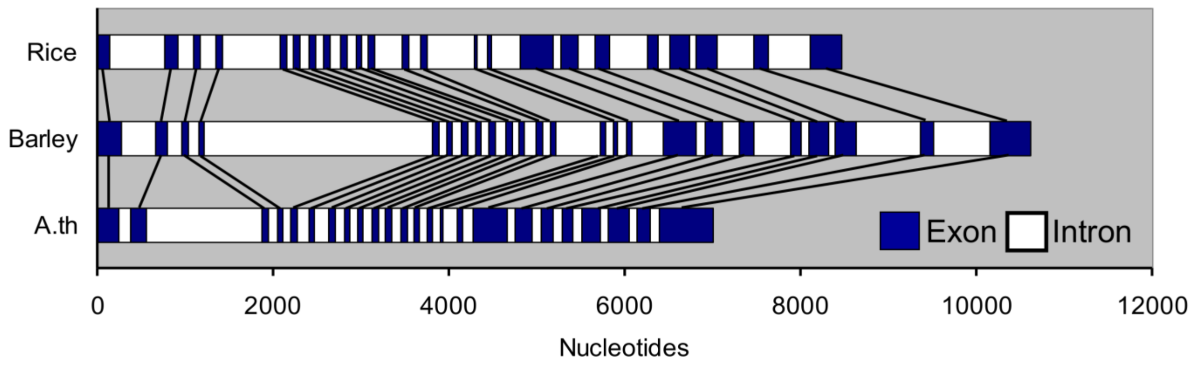

Supplementary Figure S3: Conservation of the intron/exon structure of LEMK1 genes of barley (HvLEMK1), *Arabidopsis thaliana* (At1g07650) and *Oryza sativa* (LOC Os09g17630).

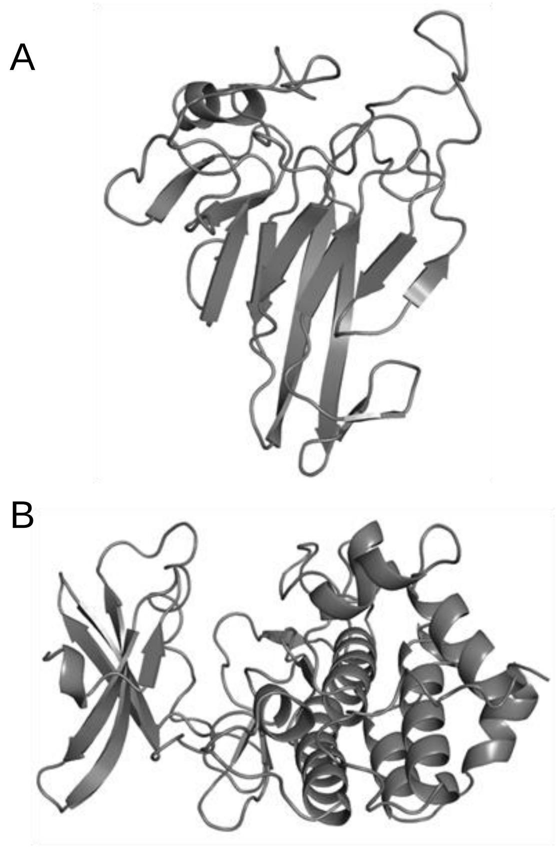

Supplementary Figure S4: 3D-models of the HvLEMK1 malectin and kinase domains.
(A) Malectin domain with the recognition site region above.
(B) The kinase domain with the active site region above, in the middle.

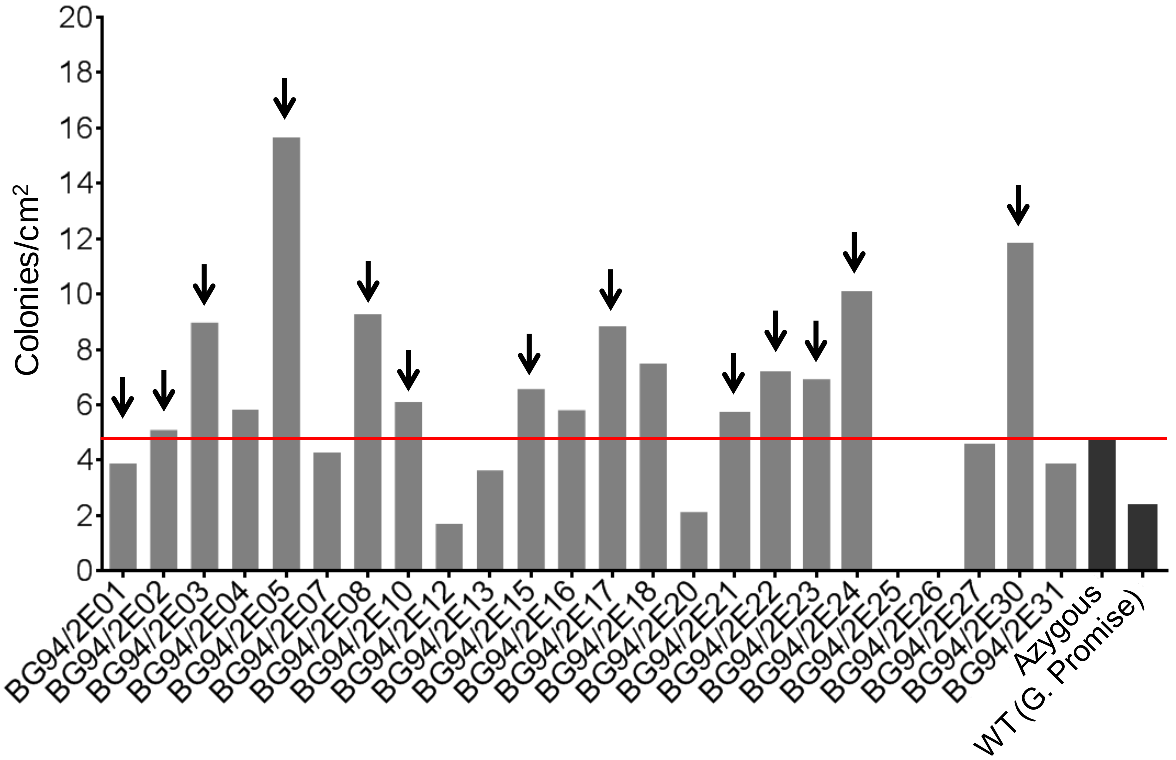

Supplementary Figure S5: Compromised NHR against *Bgt* of transgenic barley RNAi T1 families. Results from initial testing of all 24 T1 families. The red line indicates the mean susceptibility level of the pool of azygous segregant plants. Black arrows highlight primary transformation events that were analysed in repeated inoculation experiments finally resulting in four events with reproducibly increased susceptibility to the non-adapted fungus.

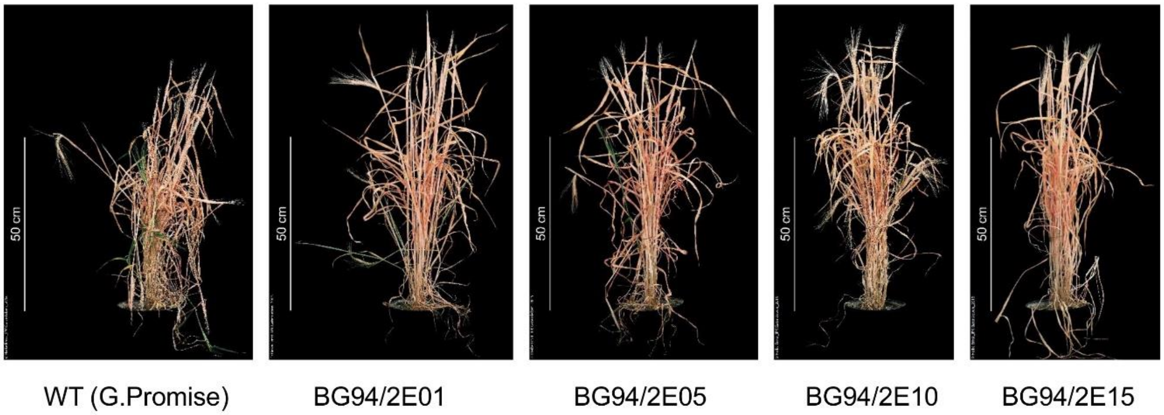

Supplementary Figure S6: No apparent growth abnormalities of T1 progeny plants silenced in Hv*LEMK1*.
Images were taken at full maturity of greenhouse-grown plants.

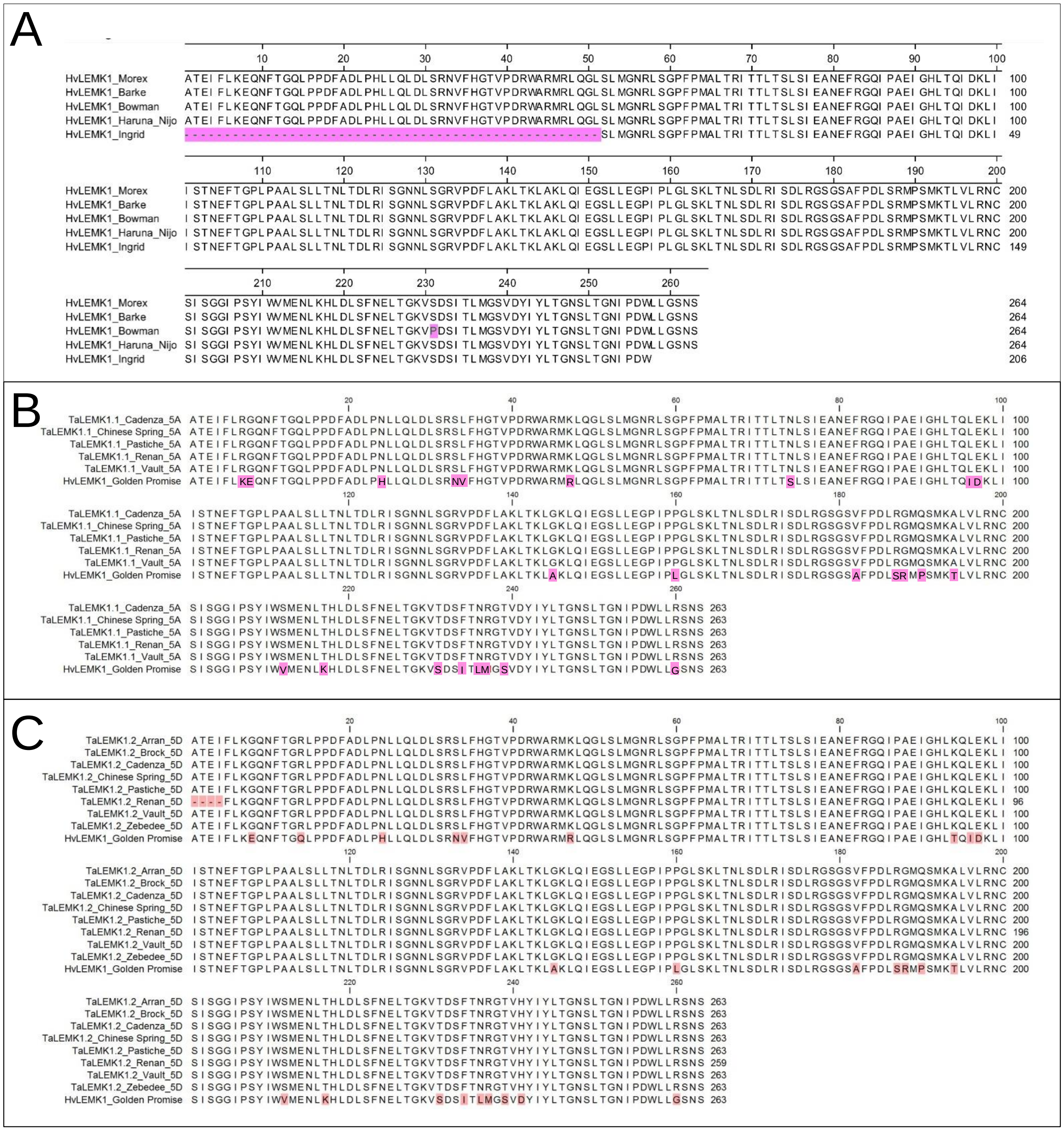

Supplementary Figure S7: Sequence alignment of *HvLEMK1* and *TaLEMK1* LRR domain from different genotypes of barley and wheat.

(A) Alignment of orthologs in barley genotypes. The deletion at the beginning if the sequence from cv. Ingrid is due to partial CDS of the corresponding cDNA clone (HO28P17, Acc DN179870). (B) Alignment of orthologs of homeologous group A in wheat genotypes. (C Alignment of orthologs of homeologous group D in wheat genotypes. (A-C) Amino acid residues differing from the consensus are highlighed by pink background.

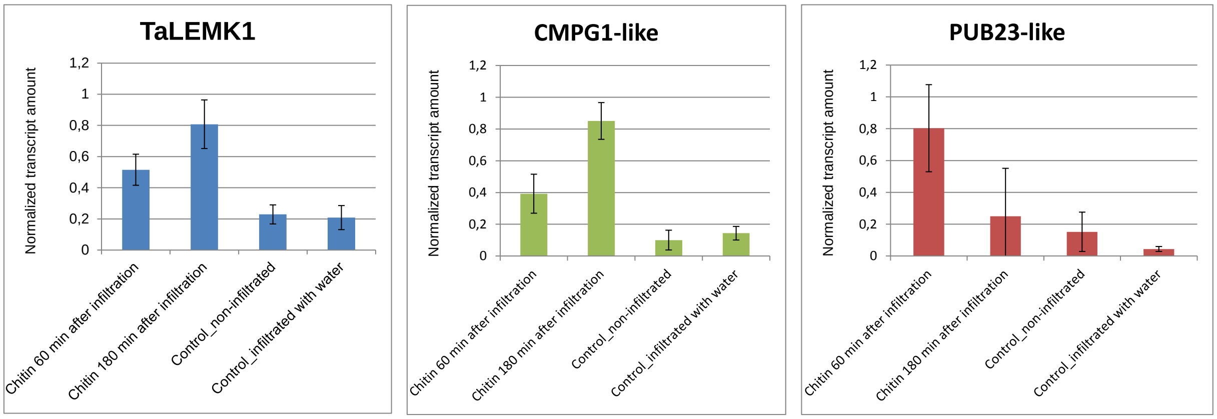

Supplementary Figure S8: *TaLEMK1* is transcriptionally up-related by the PAMP chitin.
Transcript levels of *TaLEMK1* were compared to those of the *CMPG1*-like gene involved in PAMP recognition (Gonzalez-Lamothe et al., 2006) and to the drought-stress response factor PUB23-like (Cho et al., 2015). Mean ± SEM of 5 independent biological replicates.

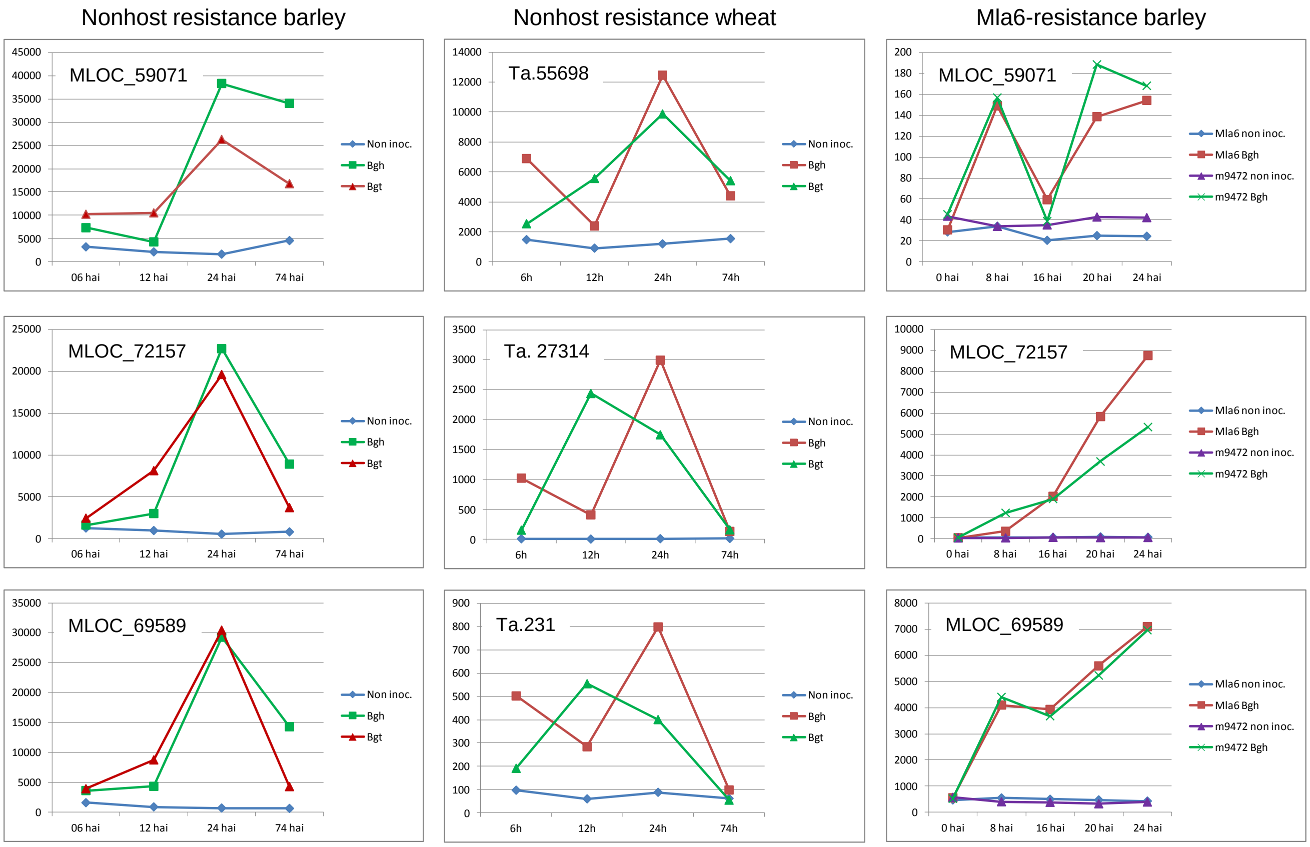

Supplemenary Figure S9: Transcript regulation of proposed PTI marker genes in nonhost- versus Mla6-resistant plants.

The data of nonhost-resistant versus host-susceptible barley and wheat are derived from 44K Agilent transcript profiling arrays (Jeyaraman, 2016) whereas the data from Mla6-resistant versus mla6 susceptible barley are derived from the Affymetrix Barley1 chip (the plant expression database

PlexDB, experiment BB10). Y axis shows quantile-normalized, log(2) transformed signal intensity values (Agilent arrays, arbitrary units) or RMA normalized data (Affymetrix chip, arbitrary units). Orthologous gene pairs of barley and wheat were determined by best reciprocal BlastN matching: MLOC_59071=Ta.55698=putative leucoanthocyanidine dioxygenase; MLOC_72157= Ta.27314=SERK1-like RLK; MLOC_69589=Ta.231=PR17-like secreted protein. Gene IDs correspond to the following probe IDs: MLOC_59071, CUST_30704_PI390587928 (Agilent) and Contig3563_at (Affymetrix); MLOC_72157, CUST_21119_PI390587928 (Agilent) and Contig3635_s_at (Affymetrix); MLOC_69589, CUST_16542_PI390587928 (Agilent) and Contig590_at (Affymetrix); Ta.55698, A_99_P259336; Ta.27314, A_99_P071865; Ta.231, A_99_P242796. Mean values from three biological replicates are shown. Expression profiles of all genes were significantly different ($p<0.05$, FDR corrected) in both nonhost versus host comparisons.

References only cited in Supplementary Figures:

Gonzalez-Lamothe R, Tsitsigiannis DI, Ludwig AA, Panicot M, Shirasu K, Jones JDG. 2006. The U-Box protein CMPG1 is required for efficient activation of defense mechanisms triggered by multiple resistance genes in tobacco and tomato. *Plant Cell* 18, 1067-1083.

Cho SK, Bae H, Ryu MY, Wook Yang S, Kim WT. 2015. PUB22 and PUB23 U-BOX E3 ligases directly ubiquitinate RPN6, a 26S proteasome lid subunit, for subsequent degradation in Arabidopsis thaliana. *Biochem Biophys Res Commun 464(4): 994-999.*
